# Supplementary material for: Peripheral Nerve Impairment in a Mouse Model of Alzheimer’s Disease
Source: Brain Sci. 2021 Sep 20;11(9):1245. doi: 10.3390/brainsci11091245 (PMC8465822; doi:10.3390/brainsci11091245)
Supplement: Supplementary file 1 [file brainsci-11-01245-s001.zip › brainsci-1351925-supplementary/Torcinaro_et_al_2021_Supplementary_revised_16th_September.pdf]

Gene name	RefSeq	Forward Sequence (5' → 3')	Reverse Sequence (5' → 3')	Product length
**Chrna1**	NM_007389.5	CAC CGC CAG CCA TCT TTA AA	TTC CTT GAT CAC CCA CTC CC	185 bp
**Chrng**	NM_009604.3	GCA GGC AGT ATT GGA GAA GC	AGG TTA CAG GCA TCC ACA CA	117 bp
**Drd1 ***	NM_010076.3	AAT CTA GGG ACC AGC GGA TG	GCA ATC CAA GCC ATA CCA GG	103 bp
**Gabbr1**	NM_019439.3	CCA CGG AGA TTG TCA TGC TG	GAA GCC TCC AGT CTC CTC AG	122 bp
**Gad1**	NM_008077.5	AAG GGC CAA TTC AGT CAC CT	CGT CAT ACT GCT TGT CTG GC	149 bp
**Tbp**	NM_013684.3	CTG GAA TTG TAC CGC AGC TT	TCC TGT GCA CAC CAT TTT TC	193 bp

Supplementary Table 1. List of primers used for qRT-PCR analysis. All the murine expression primers used in this study span an exon-exon junction. Asterisk (*) indicates murine expression primers, which do not span exon-exon junction.

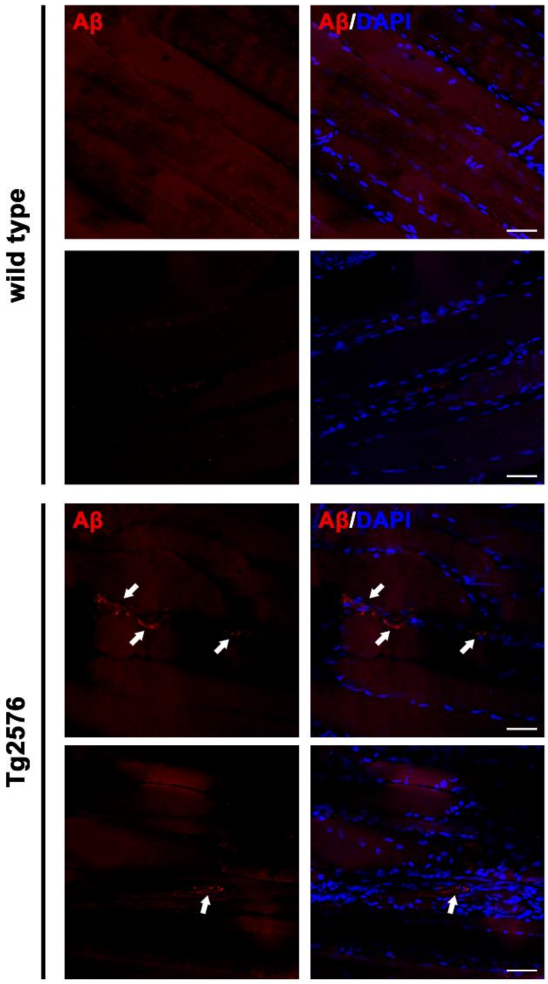

Supplementary Figure 1. Tg2576 mice accumulate human β-Amyloid peptide (Aβ) in skeletal muscle. Representative images of longitudinal TA sections, from Tg2576 and wild type mice, stained with an antibody anti-Aβ (red), which recognizes endogenous levels of total Aβ. Sections were counterstained with DAPI in order to detect cell nuclei. White arrows point to Aβ aggregates. Scale bar = 50 μm.

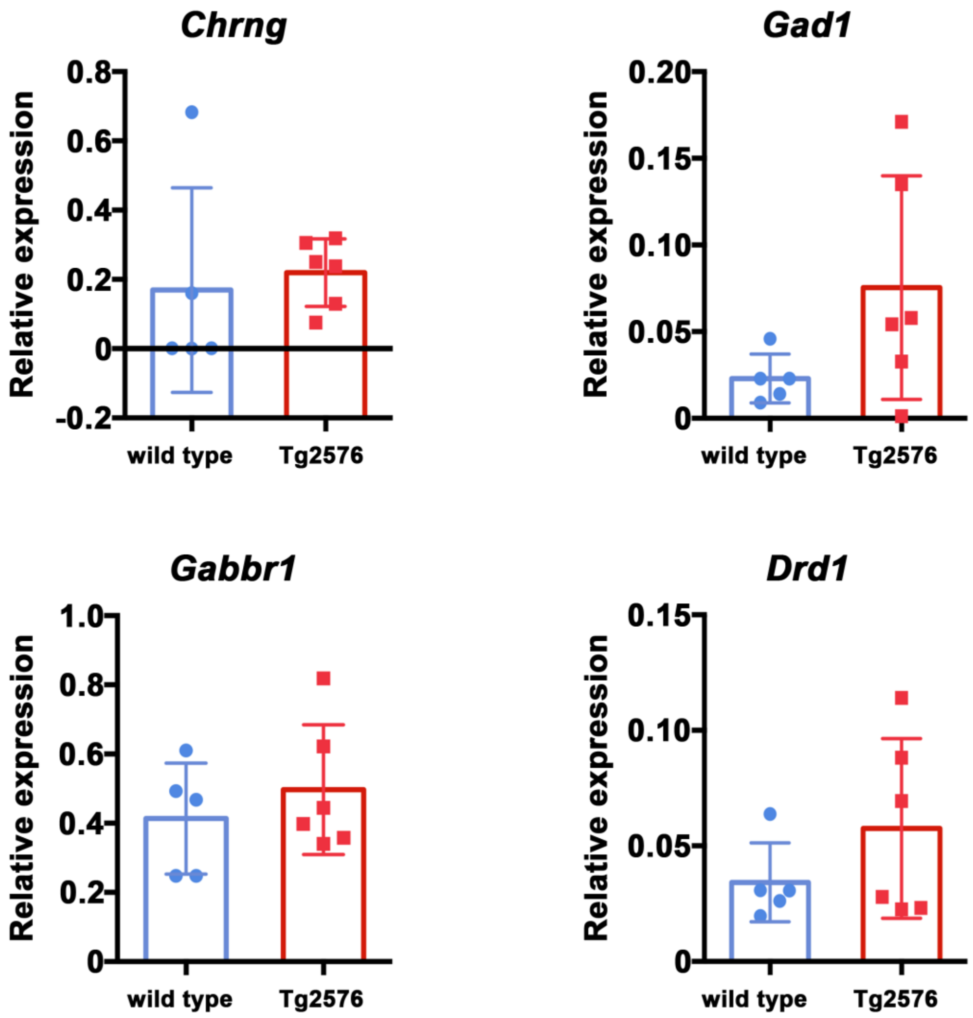

Supplementary Figure 2. Cholinergic receptor nicotinic gamma polypeptide (Chrng) and members of GABA, Glutamate and Dopamine pathways are not significantly modulated in skeletal muscle of Tg2576 mice. Expression analysis of Chrng (P=0.7011), Gad1 (P=0.1106), Gabbr1 (P=0.4537) and Drd1 (P=0.2479) by qRT-PCR, on TA muscles derived from 6-month-old female wild type (n = 5 mice) and Tg2576 mice (n = 6 mice). Data are reported as relative to housekeeping gene TBP, and represented as mean ± SEM.
